# International society of sports nutrition position stand: β-hydroxy-β-methylbutyrate (HMB)

**DOI:** 10.1080/15502783.2024.2434734

**Published:** 2024-12-19

**Authors:** John A. Rathmacher, Lisa M. Pitchford, Jeffrey R. Stout, Jeremy R. Townsend, Ralf Jäger, Richard B. Kreider, Bill I. Campbell, Chad M. Kerksick, Patrick S. Harty, Darren G. Candow, Brandon M. Roberts, Shawn M. Arent, Douglas S. Kalman, Jose Antonio

**Affiliations:** a MTI Biotech Inc, Ames, IA, USA; b lowa State University, Department of Animal Science, Ames, IA, USA; c Iowa State University, Department of Kinesiology, Ames, IA, USA; d University of Central Florida, School of Kinesiology and Rehabilitation Sciences, Orlando, FL, USA; e Research, Nutrition, and Innovation, AG1, Carson City, NV, USA; f Concordia University Chicago, Health & Human Performance, River Forest, IL, USA; g Increnovo LLC, Whitefish Bay, WI, USA; h Texas A&M University, Exercise & Sports Nutrition Lab, Department of Kinesiology and Sports Management, College Station, TX, USA; i University of South Florida, Performance & Physique Enhancement Laboratory, Exercise Science Program, Tampa, FL, USA; j Lindenwood University, Exercise and Performance Nutrition Laboratory, College of Science, Technology, and Health, Saint Charles, MO, USA; k University of Regina, Faculty of Kinesiology and Health Studies, Regina, SK, Canada; l 10 General Greene Ave, Military Performance Division, US Army Research Institute of Environmental Medicine, Natick, MA, USA; m University of South Carolina, Department of Exercise Science, Arnold School of Public Health, Columbia, SC, USA; n Dr. Kiran C Patel College of Osteopathic Medicine, Nova Southeastern University, Nutrition Department, Davie, FL, USA; o Nova Southeastern University, Department of Health and Human Performance, Davie, FL, USA

**Keywords:** β-Hydroxy-β-Methylbutyrate, sport nutrition, supplementation, leucine

## Abstract

Position Statement: The International Society of Sports Nutrition (ISSN) bases the following position stand on an analysis of the literature regarding the effects of β-Hydroxy-β-Methylbutyrate (HMB). The following 12 points have been approved by the Research Committee of the Society: 1. HMB is a metabolite of the amino acid leucine that is naturally produced in both humans and other animals. Two forms of HMB have been studied: Calcium HMB (HMB-Ca) and a free acid form of HMB (HMB-FA). HMB-FA appears to lead to increased appearance of HMB in the bloodstream when compared to HMB-Ca, though recent results are mixed. 2. The available safety/toxicity data suggest that chronic HMB-Ca and HMB-FA consumption are safe for oral HMB supplementation in humans up to at least one year. 3. There are no negative effects of HMB-Ca and HMB-FA on glucose tolerance and insulin sensitivity in humans. There may be improvements in glucose metabolism in younger adults. 4. The primary mode of action of HMB appears to be through its dual mechanism to enhance muscle protein synthesis and suppress muscle protein breakdown. HMB’s activation of mTORC1 is independent of the leucine-sensing pathway (Sestrin2-GATOR2 complex). 5. HMB may help reduce muscle damage and promote muscle recovery, which can promote muscle growth/repair. HMB may also have anti-inflammatory effects, which could contribute to reducing muscle damage and soreness. 6. HMB consumption in close proximity to an exercise bout may be beneficial to increase muscle protein synthesis and attenuate the inflammatory response. HMB can provide a beneficial physiological effect when consumed both acutely and chronically in humans. 7. Daily HMB supplementation (38 mg/kg body weight) in combination with exercise training may improve body composition through increasing lean mass and/or decreasing fat mass with benefits in participants across age, sex, and training status. The most pronounced of these improvements in body composition with HMB have been observed in studies with robust resistance training programs and dietary control. 8. HMB may improve strength and power in untrained individuals, but its performance benefits in trained athletes are mixed and increase with an increase in study duration (>6 weeks). HMB’s beneficial effects on athletic performance are thought to be driven by improved recovery. 9. HMB supplementation appears to potentially have a positive impact on aerobic performance, especially in trained athletes. The mechanisms of the effects are unknown. 10. HMB supplementation may be important in a non-exercising sedentary and aging population to improve muscle strength, functionality, and muscle quality. The effects of HMB supplementation with exercise are varied, but the combination may have a beneficial effect on the treatment of age-associated sarcopenia under select conditions. 11. HMB may be effective in countering muscle disuse atrophy during periods of inactivity due to illness or injury. The modulation of mitochondrial dynamics and lipid metabolism by HMB may be a potential mechanism for preventing disuse atrophy and aiding rehabilitation beyond HMB’s effects on rates of muscle protein synthesis and degradation. 12. The efficacy of HMB in combination with certain nutrients may be enhanced under select conditions.

## 1. Introduction

**Figure 1.**
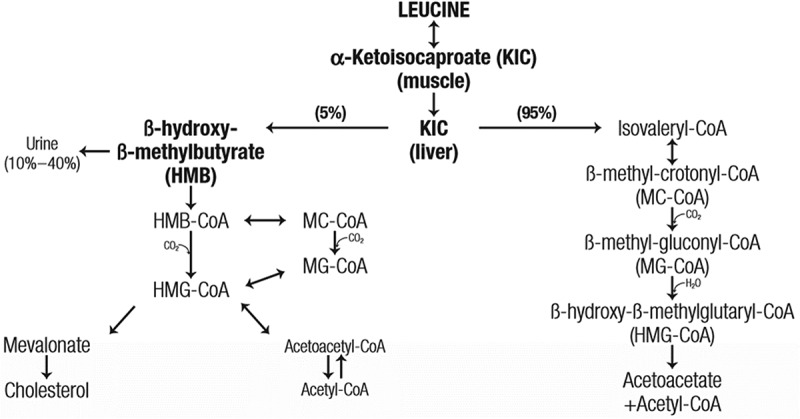
HMB (β-hydroxy-β-methylbutyrate) is the active metabolite of leucine, a branched chain amino acid. HMB is naturally occurring in humans and animals.

Beta-hydroxy-beta-methylbutyrate (HMB) is a metabolite of the amino acid leucine that is naturally produced in both humans and other animals [1] (Figure 1). The *de novo* production has been described by others [1,2], but briefly, leucine is reversibly transaminated to α-keto-isocaproate (KIC) by branched-chain amino acid transferase extrahepatically. KIC can be metabolized to either isovaleryl-CoA (by α-ketoacid dehydrogenase) in the mitochondria by the α-ketoacid dehydrogenase or HMB (by α-ketoisocaproate dioxygenase) in the cytosol. As most KIC is metabolized into isovaleryl-CoA, only ~ 5% of leucine is converted into HMB [1,3–5]. HMB has been studied in humans for nearly three decades [1,6,7] at doses ranging from 1.5 g/d to 6 g/d [6,7] across a variety of populations and situations. Since the first publication by Nissen et al. [6], HMB has been studied in a variety of exercise training conditions in both the young and the old, and these studies support the efficacy of HMB supplementation for enhancing recovery, lean body mass (LBM), muscle strength and power, and aerobic performance. Herein, we provide an analysis of the HMB literature with a particular focus on new research published since the original International Society of Sports Nutrition (ISSN) position stand on HMB published over a decade ago [8].

## 2. Methods

ISSN position stands are invited papers of topics the ISSN editors and Research Council identify as topics of interest to our readers that need position stands to provide guidance to readers and the profession. Editors and/or the Research Council identify a lead author or team of authors to perform a comprehensive literature review. The draft is then sent to leading scholars for review and comment. The paper is then revised as a consensus statement and reviewed and approved by the Research Council and Editors as the official position of the ISSN.

The authors utilized a scoping review of the scientific literature related to the forms, safety, mode of action, physiological effects and nutritional aspects of HMB. The authors accessed a database of over 750 original articles and reviews maintained through PubMed, Google Scholar, and ResearchGate database searches by using the following terms: HMB, beta-hydroxy-beta-methylbutyrate, beta-hydroxy-beta-methylbutyric acid, 3-hydroxy-3-methylbutyrate, and beta-Hydroxyisovaleric acid. The expert author group identified and provided any additional papers that they deemed to have been missed in the literature searches.

## 3. Forms of HMB

As a dietary supplement, all of the studies published until 2011 utilized the calcium salt of HMB (HMB-Ca). Fuller et al. [9] in 2011 were the first to describe a free acid form of HMB (HMB-FA). Though most clinical studies of HMB have evaluated the effects of HMB-Ca, HMB-FA may provide a more efficient HMB delivery option. To date, three studies have directly compared the kinetics of HMB-Ca and HMB-FA in humans [9–11]. In the first study, an HMB-equivalent dose of HMB-Ca was compared to an oral or sublingual HMB-FA gel and resulted in earlier and higher peaks in plasma HMB [9]. HMB-FA administration also led to a 15% greater increase in the plasma HMB area under the curve (AUC) relative to HMB-Ca without notable changes in urinary losses over 24 hours, resulting in ~ 25% increased clearance and apparent utilization of the HMB dosage [9]. Similar results were observed when comparing HMB-Ca to HMB-FA when delivered in multiple formulations (as a gel, a hard gelatin capsule, or dissolved in water) [10]. Regardless of delivery format, HMB-FA was found to be more readily available and had a higher clearance compared with HMB-Ca. However, Ribeiro and colleagues [11] recently reported conflicting results, finding that HMB-Ca administered via capsule or dissolved in water resulted in significantly higher plasma HMB concentrations, faster time to peak concentration, greater AUC, and improved bioavailability compared to HMB-FA administered in a soft gel veg-capsules. The type of capsule differed from a previous study [10]. Though both forms have been shown to be effective, the earlier peak in HMB levels with HMB-FA reported by the first two investigations may support strategic timing of HMB delivery during optimal nutrition windows. However, few clinical studies have directly compared the clinical effects of supplementation with HMB-Ca vs HMB-FA [12,13], and none have specifically evaluated the timing of HMB administration on outcomes, therefore, additional research is needed.

In summary, two forms of HMB have been studied: HMB-Ca and a free acid form of HMB, HMB-FA. HMB-FA appears to lead to increased appearance of HMB in the bloodstream when compared to HMB-Ca, though recent evidence is mixed.

## 4. Safety and toxicity of HMB-Ca and HMB-FA

The safety and toxicity of HMB-Ca and HMB-FA has been extensively studied *in vitro* [14], in animals [1,15–17] and in humans [1,18–23] over the last 30 years. The previous ISSN HMB position stand concluded that chronic supplementation with HMB was safe for both young and older populations [8]. Several toxicity studies have been published for HMB-Ca, including acute [15] and long-term toxicity studies in rats [17], dosing in large animals [1]. Additionally, Pitchford et al. [14] reported the results of a battery of genotoxicity studies. None of these studies reported any genotoxicity from HMB-Ca.

An acute dosing study per OECD (Organization for Economic Co-operation and Development 420) guidelines was carried out in rats given a single oral dose of 2,000 mg/kg body weight of HMB-Ca and monitored for 14 days. There were no deaths or significant adverse clinical signs reported and it was concluded that HMB is nontoxic [15]. Similarly, the acute effects of HMB-Ca were examined in young pigs and lambs that were fed HMB-Ca per day for four days [1]. Blood chemistry and hematology, organ pathology, and histology were not affected. Additional longer studies were carried out in rats fed diets containing 1%, 2%, or 5% food grade HMB-Ca or a control diet for 91 days, in compliance with the Food and Drug Administration (FDA) and GLP (Good Laboratory Practice) regulations [17]. These showed no HMB-Ca-related adverse effects on clinical signs, body weight, feed consumption, clinical chemistry, hematology, organ weights, or tissue observations. The study established no-observed-adverse-effect-levels (NOAEL) of 3.49 and 4.16 g·kg BW^−1^·d^−1^ in male and female rats, respectively. Based on the FDA recommended body surface area correction factors [24,25], these levels translate to ∼0.5 g·kg BW^−1^·d^−1^ in humans, corresponding to more than 30 g/d for a 60-kg person, which is more than 10 times the recommended dose. A more recent 90-day rat toxicity study of supplementation with the free acid form of HMB (HMB-FA) yielded similar results, with no toxicologically relevant observations at HMB-FA consumption levels of up to 4% in the diet [16], which corresponded to an intake of 2.48 and 2.83 g·kg BW^−1^·d^−1^ in male and female rats, respectively.

In terms of toxicity, HMB-Ca and HMB-FA have a strong safety profile in humans. Consuming up to 6 g of HMB-Ca per day up to 8 weeks did not lead to any changes in blood chemistries or the biochemical parameters of renal function, hepatic function, or hematology [19,26]. Nissen et al. [18], reviewed safety data from nine clinical studies lasting between 3 and 8 weeks, which included both young and older adults. No adverse events, either clinical or laboratory-based, were reported. However, there was a decrease in total cholesterol by 5.8%, LDL cholesterol by 7.3%, and an average decrease in systolic blood pressure by 3.3%. These results indicate that HMB-Ca is safe for use in the general population and provides some cardiovascular health benefits. A similar safety profile was observed in a 12-week study of HMB-FA [27].

Additional safety data were collected by Rathmacher et al. [23] examining the effects of daily dosing with a mixture of HMB-Ca, arginine, and glutamine in healthy adult males, as well as in HIV-positive patients with HIV associated weight loss and cachectic cancer patients. Treated subjects showed no clinical, hematological, or biochemical laboratory adverse events in any of these studies. Two year-long studies have been carried out in elderly men and women receiving a mixture of HMB-Ca with arginine and lysine [22] or HMB-Ca plus vitamin D_3_ [21]. These long-term supplementation studies were not associated with any adverse events or changes in renal function, hepatic function, or hematology.

Although previous human studies found no negative effect of HMB-Ca or HMB-FA on fasting blood levels of glucose and insulin, glucose tolerance, or insulin sensitivity [18,20,28–30], studies in rats showed varied effects. Some rodent studies have observed potential negative effects of HMB on glucose tolerance and/or insulin sensitivity [31–33]. Nunes et al. [31] studied the effects of HMB-Ca supplementation (320 mg·kg^−1^· d^−1^) in rats exposed to glucocorticoid excess and showed that the HMB-Ca-supplemented group had mildly (<10 mg/dL) elevated fasting blood glucose levels and poorer glucose tolerance than the rats receiving only dexamethasone; importantly, HMB-Ca treatment alone did not affect any measures of insulin responsiveness. In a study by Yonamine et al. [32], sedentary rats given HMB (320 mg·kg^−1^·d^−1^) for four weeks demonstrated worsened glucose and insulin responsiveness over a 30-minute intravenous glucose tolerance test. A recent study by Schadock et al. [33] found that HMB-Ca had mixed effects on measures of glycemia and insulin sensitivity in mice. Fasting glucose was mildly (~12 mg/dL) higher in trained, but not untrained, mice supplemented with HMB-Ca. Interestingly, HMB improved glucose tolerance only in untrained mice, while this effect was not observed in trained mice; glucose tolerance was equivalent in trained mice regardless of HMB supplementation. In contrast, well-controlled rodent toxicity studies found no effects of HMB-Ca or HMB-FA supplementation on glycemia [16,17]. Another study by Sharawy et al. [34] found that HMB-Ca supplementation in rats improved fasting insulin levels, attenuated high-fructose-diet-induced insulin resistance, and improved HOMA-IR index, and HbA1c. HMB-Ca supplementation also improved fasting glucose and insulin levels in a rat tumor model [35]. In another study, HMB-Ca supplementation did not alter blood glucose and insulin levels, HOMA-IR index, or muscle glucose uptake in diet-induced obese C57BL/6 mice [36].

The data in humans differ from the varied effects observed in rodent models. In humans, Wilkinson et al. [28] showed that oral ingestion of 2.42 g HMB-FA in healthy young men did not alter circulating fasting or postprandial insulin levels, but leucine did, suggesting HMB is not an insulin secretagogue. Yet HMB-FA decreased muscle protein breakdown (−57%) in a fashion similar to, but independent of insulin. Townsend et al. [29] found that HMB-FA consumption (1 g) by young, resistance-trained men did not affect serum insulin levels before, immediately after, or 30 minutes after acute heavy resistance exercise. Interestingly, when HMB is combined with glucose the results may change. For example, a study by Herrod et al. [37] evaluated the impact of a 3-g ingestion of HMB-Ca in humans during an oral glucose tolerance test (OGTT) and found a reduction in insulin secretion in young men, but not older men. However, another study found the opposite with HMB-FA, indicating it has no impact on postprandial glucose or insulin during an OGTT. The differences between these studies could be due to the type of HMB used, the timepoints analyzed, or the difference in analytical techniques.

In summary, the available data suggests that long-term oral HMB supplementation (e.g. 1.5–3 g/d) is safe for humans for at least one year in length. Short-term dosing up to 6 g/d for 8 weeks has no adverse effects. Further research is needed to determine the role of chronic HMB supplementation on insulin sensitivity and glucose metabolism with consideration for age and sex.

## 5. Hmb’s primary mode of action

Changes in muscle mass are regulated by the balance of muscle protein synthesis and muscle protein breakdown [38]. When the rate of muscle protein synthesis exceeds the rate of muscle protein breakdown, there is a net increase in muscle protein. Conversely, when the rate of muscle protein breakdown exceeds the rate of muscle protein synthesis, there is a net decrease in muscle protein. The primary and most investigated mode of action of HMB has been through its dual mechanism to enhance muscle protein synthesis and suppress muscle protein breakdown.

Like its parent amino acid, leucine, HMB upregulates muscle protein synthesis via the mammalian target of rapamycin (mTOR) and its downstream targets ribosomal protein S6 kinase (p70S6K1) and eukaryotic initiation factor-4 binding protein-1 (4EBP1) *in vitro* [39]. It is thought that leucine metabolites, such as HMB, may contribute to or directly drive the anabolic responses to leucine because leucine is metabolized within the muscle. *In vitro* evidence suggests that the conversion of leucine into HMB is necessary for the maximal stimulation of protein synthesis [40]. Furthermore, Suryawan et al. [41] demonstrated that both leucine and HMB stimulate the mechanistic target of rapamycin complex 1 (mTORC1) phosphorylation in muscle. Leucine’s action involves the dissociation of the Sestrin2-GATOR2 complex and increased binding of Rag A/C to mTOR, whereas, HMB’s activation of mTORC1 is independent of this leucine-sensing pathway. Clinical study results show that a 3 g dose of HMB induces a robust (near-maximal) stimulation of muscle protein synthesis in human muscle via activation of mTORC1 and downstream phosphorylation of p70S6K1, in agreement with *in vitro* evidence [28,42]. This stimulation is independent of the HMB form, with similar results observed for HMB-Ca and HMB-FA in separate studies [28,42]. As HMB supplementation can increase growth hormone (GH) and insulin-like growth factor 1 (IGF-1) levels [29], it may also stimulate protein synthesis via GH/IGF-1 axis signaling, though HMB-induced increases in these hormones have not been directly linked to protein synthesis. Some studies have proposed that HMB stimulation of muscle protein synthesis involves activating AKT [43–45]. However, others have shown *in vivo* [46,47] that HMB enhances muscle protein synthesis without activation of AKT. Additionally, HMB has been shown to activate AMP activated protein kinase (AMPK) *in vitro* [48].

Preclinical evidence shows that HMB also decreases muscle protein breakdown through multiple pathways, including the suppression of the ubiquitin-proteasome pathway [49–51], inhibition of myonuclear apoptosis via mitochondrial-associated caspase signaling [52], and suppression of lysosomal autophagy pathways [53]. Clinical studies show that 3 g of HMB significantly decreases muscle protein breakdown independent of HMB form (i.e. calcium vs free acid) [28,42]. HMB-Ca supplementation during resistance training also results in dose-dependent decreases in muscle protein breakdown as measured by urinary 3-methylhistidine, a byproduct of muscle contractile protein breakdown that is not metabolized or re-used for protein synthesis [6]. Though muscle protein breakdown is typically regulated through an insulin-dependent process [54], HMB-FA suppresses muscle protein breakdown in human muscle via an insulin-independent process [28].

The specific mechanisms by which HMB suppresses muscle protein breakdown in humans remain to be elucidated. The primary clinical evidence of HMB’s mechanisms of action on protein synthesis and protein breakdown, though methodologically rigorous, is limited by the inclusion of only young, healthy men in these studies. No changes in the expression of atrophy-associated proteins (e.g. MuRF1, MAFbx) were observed in human muscle during clinical studies, though the researchers noted that peak signaling events may have occurred outside the single timepoint evaluated [28,42]. Baier et al. [22] evaluated rates of whole-body protein turnover by using primed/intermittent oral doses of ^15^N-glycine. In older adults, a combination of HMB-Ca with arginine and glutamine increased rates of protein turnover at 3 and 12 months of supplementation, respectively. A more recent study examined muscle protein synthesis during 6 weeks of single-leg resistance exercise training with or without HMB in free-living older men using deuterium oxide methods [55]. While no main effects of HMB supplementation alone on muscle protein synthesis were observed, during the first two weeks of training, a significant increase in muscle protein synthesis with exercise was observed in the HMB group. The meaningfulness of these findings (or lack thereof) from this study is undermined by the limited muscle mass exercised and the inability to measure muscle protein breakdown. To our knowledge, other potential mechanisms for reduced muscle protein breakdown have not been evaluated in clinical studies. Additional research in women and older populations will add valuable information to our understanding of HMB’s effects across different populations.

In summary, the primary mode of action of HMB appears to be through its dual mechanism to enhance muscle protein synthesis and suppress muscle protein breakdown.

## 6. Effects of HMB on muscle damage and functional recovery

Data suggests that HMB may help reduce muscle damage and promote muscle recovery. Indeed, two studies support the hypothesis that HMB supplementation helps prevent exercise-induced muscle damage and improves recovery following exercise [56,57]. Supplementation with 3 g of HMB-Ca resulted in a decreased creatine phosphokinase (CK) and LDH response after a prolonged run [57], and HMB-Ca protected against the exercise-induced rise in CK in men [56] in response to progressive resistance-exercise training. Panton et al. [58] reported that men and women who supplemented for four weeks with 3 g HMB-Ca in conjunction with progressive resistance training saw a significantly decreased CK response following the intervention. Furthermore, compared to a placebo group, 14 days of HMB-Ca supplementation (3 g/d) prior to an eccentric bicep curl exercise reduced the subsequent CK elevation, which corresponded with better maintenance of 1-repetition maximum (1-RM) curl strength and reduced soreness compared to placebo during a 72- hours of recovery [59]. Additionally, a muscle-damaging bout of isokinetic, eccentric knee extensor and flexor exercise was performed by 16 untrained participants who were given 3 g of HMB-Ca 60 minutes before or immediately after exercise. In contrast to the post-exercise feeding group, those who supplemented with HMB pre-exercise showed lower elevations in LDH in recovery [60]. Recently, Tsuchiya et al. [61] supplemented untrained males with 3 g/d of HMB-Ca for 2 or 4 weeks before an eccentric upper-body muscle damaging protocol. The results indicated that both 2 and 4 weeks of HMB supplementation improved the recovery of MVC torque and range of motion compared to a placebo while reducing muscle stiffness and swelling. Moreover, while most investigations to date utilized a 3 g/d dose of HMB, a subsequent study from these researchers investigated the efficacy of a 1.5 g/d HMB dose taken for two weeks before eccentric damage to the elbow flexors. Interestingly, the low dose of HMB was also efficacious for improving functional recovery, including improvements in maximal voluntary contraction and range of motion of the elbow flexors in these untrained males [62].

While some protective effects of HMB have been shown in untrained participants, current data are mixed regarding resistance-trained individuals. One study in NCAA Division I football players found no effect of 28 days of HMB supplementation on markers of muscle damage or measures of strength and power performance [19]. Hoffman et al. [63] investigated whether HMB-Ca could provide a protective effect during a pre-season summer football training camp, which commonly consists of two practices per day. Results from this study indicated HMB had no benefit regarding physical performance, CK, myoglobin, or hormonal status. Some have postulated that since resistance-trained athletes have acquired more protection against muscle damage via the repeated bout effect, these individuals may need to be subjected to a profound amount of stress or training to experience an ergogenic effect from HMB. As such, Wilson et al. [27] provided 3 g/day of HMB-FA or placebo to trained males before and for two days after a strenuous lower body resistance exercise session. At 48-hours post-exercise, CK levels were significantly higher in the placebo group while perceived recovery was greater in the HMB-FA group. However, when utilizing a similar supplemental dose of HMB-FA, no reduction in inflammatory cytokines, CK, myoglobin, C-reactive protein, or squat performance 24 and 48 hours following the muscle-damaging bout of exercise was seen [64]. Correia et al. [65] found that HMB-FA improved the recovery of exercise capacity, as measured by 30 maximal isokinetic knee extensions at 120º/s, 24 hours following seven sets of 20 muscle-damaging drop jumps. Several other studies in competitive athletes have shown promising effects of HMB on indirect markers of muscle damage. Eslami et al. [66] saw that HMB attenuated CK and LDH responses when taken during an intense training period in competitive soccer players, and these findings were supported in a group of professional soccer players taking 3 g of HMB-FA [67]. In trained wrestlers, HMB-FA attenuated CK and LDH following a simulated wrestling protocol while improving metrics of perceived recovery compared to a placebo [68]. A recently published meta-analysis [69] pooled the results from 10 randomized controlled trials with 324 total participants examined the impact of HMB supplementation on muscle damage markers, finding that when 3 g of HMB per day is supplemented for longer than six weeks, HMB significantly attenuated CK and LDH responses following muscle-damaging exercise. However, additional studies comparing HMB-Ca to HMB-FA form are needed to determine if there are any differences.

## 7. Acute dosing of HMB

Several investigations have studied the effects of HMB when taken acutely as opposed to after chronic loading periods. Wilkinson et al. [28] reported significant increases in myofibrillar muscle protein synthesis (MPS) following consumption of a 3.42 g dose of HMB-FA, which increased intramuscular anabolic signaling (mTOR, p70s6k) comparable to 3.42 g of leucine in healthy males. HMB-FA stimulated MPS to a similar extent to leucine, but HMB-FA was also found to decrease muscle protein breakdown when taken acutely. Later, a bolus of 3 g of HMB-Ca was also shown to increase mTORc1 signaling and MPS rates in a study by the same group [42]. When taken before a workout, Townsend et al. [29] gave only 1 g of HMB-FA to trained males before a heavy lower body workout consisting of the squat, deadlift, and split squat, and found increased IGF-1 and GH plasma concentrations measured at 30 min and & 1 hour post exercise. Thus, HMB-FA may alter the GH/IGF-1 axis through similar pathways as arginine and lysine [70–72]; leucine has also been demonstrated to stimulate modest increases in GH concentrations [73].

Regarding immune responses, a higher percentage of monocytes expressing the complement receptor type 3 (CR3) along with elevated macrophage inhibitory protein-1β (MIP-1β) were seen following ingestion of 1 g of HMB-FA in a high-intensity resistance exercise protocol with or without cold-water immersion (CWI) therapy post workout [74]. This marker (CR3), measured by flow cytometry, specifies monocytes that enter the site of muscle damage to begin repair. These data may suggest that HMB-FA alters immune cell mobilization and adhesion mechanisms during tissue repair and recovery. Furthermore, using the same dose of HMB-FA and exercise protocol, HMB-FA and HMB-FA + CWI decreased expression of tumor necrosis factor receptor-1 (TNFR1), and attenuated tumor necrosis factor alpha (TNF-α) cytokine levels in the HMB-FA fed groups [75]. In summary, it appears that HMB can provide a beneficial physiological effect when consumed both acutely and chronically in humans.

## 8. The effect of HMB with exercise on body composition

In Table 1, the published literature evaluating the combined effects of HMB and exercise on body composition (i.e. lean mass, fat mass, or their ratio) in placebo-controlled studies without subjective bias for study design or population is available. However, the population factors, as well as other differences between study designs, likely influence the findings. HMB supplementation has been studied across a wide range of populations with regard to age, sex, and training status and has been studied in combination with a variety of training stimuli in terms of mode, duration, and intensity.

**Table 1.** Summary of placebo-controlled studies evaluating the effects of HMB and exercise on body composition.

Study	Design	Subjects	Exercise Training	Diet control or assessment	HMB duration, dose, and type	Additional supplements	Body Composition Measure	Findings (relative to placebo)
Nissen et al. [6] (Study 1)	RCT, DB	Young untrained men; 19–29 y; *n* = 6–8/group	Monitored high-intensity progressive resistance training; 3 ×/wk;	Control	3 wk; 1.5 or 3 g/d HMB-Ca	37 g milk protein in some groups	TOBEC	FFM: +0.4 (1.5 g); +0.8 (3.0 g) (*p* < 0.11) FM: NS
Nissen et al. [6] (Study 2)	RCT, DB	Young, trained men; 19–22 y; *n* = 16/group	Monitored progressive resistance training; 6 d/wk, 2–3 h/d, aerobic training ≥ 3 d/wk	No	7 wk; 3 g/d HMB-Ca	37 g milk protein	TOBEC	FFM: ~ +1.9 kg (days 14–39; *p* < 0.05) FM: NS
Kreider et al. [19]	RCT, DB	Young resistance- trained; 25.1 ± 1 y; *n* = 12–15/group	Not monitored; instructed to maintain current individualized training program; 6.9 ± 0.5 hr/w	Assessment	28 d; 3 or 6 g/d HMB-Ca	81 g carbohydrate, 75 g protein, 3 g fat	DXA	LBM: NS FM: NS
Gallagher et al. [7]	RCT, DB	Young untrained men; 18–29 y; *n* = 11–14/group	Monitored progressive resistance training; 3 d/wk	Assessment	8 wk; 38 or 76 mg/kg/d (~3 or 6 g/d) HMB-Ca	No	7-site skinfold	FFM: +1.9 kg in 38 mg/kg/d group (*p* < 0.05) FM: Not reported
Panton et al. [58]	RCT, DB	Young-to-middle-aged men and women (mixed training status); 20–40 y; *n* = 18–21/group	Monitored high-intensity progressive resistance training 3 d/wk	No	4 wk; 3 g/d HMB-Ca	No	Skinfold and underwater weighing	FFM: +0.5 kg (*p* = 0.08) %BF: −0.6% (*p* = 0.08)
Jowko et al. [56]	RCT, DB	Young active but untrained men; 19–23 y; *n* = 9–11/group	Monitored progressive resistance training 3 d/wk;	Assessment	3 wk; 3 g/d HMB-Ca	No^a^	BIA	FFM: +0.4 kg with HMB alone (*p* = 0.08) FM: NS
Slater et al. [76]	RCT, DB	Young highly trained water polo players and rowers; 24.5 ± 1.7 y; *n* = 9/group	Non-controlled workouts assigned by athletes’ coaches; 2–3 d/wk, plus sport-specific training	Assessment	6 wk; 3 g/d HMB-Ca	24 g carbohydrate; 42 g protein; 2 g fat	DXA	LBM: NS FM: NS
Vukovich et al. [77]	RCT, DB	Older untrained men and women; 70 ± 1 y; n = 14–17/group	Supervised; resistance training 2 d/wk; walking and stretching 3 d/wk for 60 min	None	8 wk; 3 g/d HMB-Ca	No	Skinfold, DXA	Skinfold: FFM: +1.0 kg (*p* = 0.08) %BF: −1.3% (*p* < 0.05) CT: Fat, ~ −8.0% DXA: NS
Ransone et al. [78]	Crossover; DB	Young male college football players; 19.2–21.3 y; *N* = 35	Supervised progressive resistance and endurance exercise; 4 d/wk; 4 h/d	No	4 wk; 3 g/d HMB-Ca	No	Skin folds	NS
Zajac et al. [79]	RCT; blinding not described	Young male trained basketball players 25.6 ± 5.6 y n = 12–23/group	Resistance training 3 d/wk	No	30 days; HMB (dose and type not specified)	No^a^	BIA	FFM: ~ + 2 kg (*p* = .05) %BF: ~ −1.3% (*p* = 0.001)
Lamboley et al. [80]	RCT; DB	Young, untrained men and women; 23 ± 1y n = 8/group	Supervised high-intensity interval training (treadmill) 3 d/wk	No	5 wk; 3 g/d HMB-Ca	No	DXA	NS
Kraemer et al. [81]	RCT; DB	Young, recreationally active but untrained men; 22.9 ±- 3.8 y; n = 8–9/group	Periodized progressive resistance training 3 d/wk; >30 min endurance exercise 2–3 d/wk	Control	12 wk, 3 g/d HMB-Ca	14 g arginine; 14 g glutamine; 6 g taurine; 11.6 g dextrose	DXA	LBM: ~+ 5 kg (*p* ≤ 0.05) %BF: ~ −2% (*p* ≤ 0.05)
Thompson et al. [82]	RCT; DB	Young, trained men; 24 ± 4 y n = 9–13/group	Progressive resistance training; 3 d/wk	Assessment	9 wk; 3 g/d HMB-Ca	No	Skinfold; BIA	Skinfold (sum): −9 units (*p* = 0.05) BIA: NS
Portal et al. [83]	RCT; DB	Elite male and female adolescent volleyball players; 13.5–18 y; n = 14/group	Combination training (intervals, power/speed, skills, progressive resistance training, endurance training); 18–22 h/wk	Assessment	7 wk; 3 g/d HMB-Ca	No	Skinfold	FFM: + 2.4 kg (*p* = .00) %BF: − 1.9% (*p* = 0.04)
Stout et al. [84]	RCT; DB	Healthy untrained older men and women; (73 ± 1 y); n = 16–20/group	Supervised progressive resistance training; 3 d/wk	Assessment	24 wk; 3 g/d HMB-Ca	No	DXA	LBM: −0.2 kg in men (*p* = 0.03)
^c^Wilson et al. [85]	RCT; DB	Young resistance-trained men; 20–28 y; n = 9–11/group	Supervised periodized resistance training with 2-wk overreaching cycle; 3–5 d/wk	Control	12 wk; 3 g HMB-FA	No	DXA	LBM: + 5.3 kg (*p* = .001) FM: − 3.7 kg (*p* = 0.0003)
Stout et al. [86]	RCT; DB (post hoc analysis of Stout 2013)	Healthy untrained older men; 72.1 ± 5.7 y; n = 12/group	Supervised progressive resistance training; 3 d/wk	Assessment	12 wk; 3 g/d HMB-Ca	No	DXA	Abdominal adiposity: −0.22 kg (*p* < 0.05)
Durkalec Michalski et al. [87]-	RCT; DB; crossover	Young elite male rowers; 17–22 y; n = 16	Continue usual rowing training (10–24 h/wk)	Assessment	12 wk; 3 g/d HMB-Ca	No	BIA	FFM: NS FM: −1.7 kg (*p* = 0.003)
Durkalec-Michalski et al. [88]	RCT; DB; crossover	Young, trained combat sport athletes; 22.8 ± 6.1 y n = 42	Continue usual sport, endurance, and strength/power training (8.3 sessions/wk)	No	12 wk; 3 g/d HMB-Ca	No	BIA	FFM: + 1.5 kg (*p* = .049) FM: −1.5 kg (*p* = 0.016)
McIntosh et al. [89]	RCT; DB	Young male rugby players; 18–27 y; n = 11–12/group	Rugby training program including progressive resistance training 4 d/wk and sport-specific training 4 d/wk	Assessment	12 wk; 3 g/d HMB-Ca	No	Skinfold	%BF: NS
Teixeira et al. [90]	RCT; DB	Young to middle-aged trained men; 31.7 ± 7.6 y; 9–11/group	Progressive resistance training	Assessment	8 wk; 3 g/d HMB-Ca or HMB-FA	No	DXA	NS
Tinsley et al. [91]	RCT; DB	Young, trained women; 18–30 y; n = 13–14/group	Supervised progressive resistance training 3d/wk	Control (time-restricted feeding)	8 wk; 3 g/d HMB-Ca	25 g whey protein	Modified 4C model produced from DXA and BIS	ITT analysis: NS PP analysis: FFM: NS FM: − 0.6 (*p* = .03) %BF: −0.9% (*p* = 0.048)
Tritto et al. [12]	RCT; DB	Young, trained men; 25.3 ± 3.7 y; n = 15/group	Progressive resistance training 2 d/wk	Assessment	12 wk; 3 g/d HMB-Ca or HMB-FA	No	DXA	NS
Fernández-Landa et al. [92]	RCT; DB	Young elite male rowers; 30.4 ± 4.7 y; n = 7/group	Continued rowing practice 6 d/wk	Control (personalized)	10 wk; 3 g/d HMB-Ca	1 g/kg carbohydrate; 0.3 g/kg protein^a^	Skinfold	NS
Rathmacher et al. [21]	RCT; DB	Older untrained men and women; > 60 y; n = 26–34/group	Supervised moderate-intensity progressive resistance training 3 d/wk	Assessment	12 months; 3 g/d HMB-Ca	2000 IU vitamin D3	DXA	NS
Stahn et al. [93]	RCT; DB	Young untrained men; 19–25 y; n = 7–8/group	Supervised periodized resistance training 4 d/wk	No	12 wk; 3 g/d HMB-Ca	30 g whey protein daily; additional 30 g whey protein and 30 g carbohydrate supplement on training days	BIS	NS interaction; medium-to-large effect sizes for greater effect of HMB on total/segmental FFM
Osuka et al. [94]	RCT; DB	Older women with low muscle mass; 65–79 y; n = 39/group	Supervised progressive resistance training 2×/wk	Assessment	12 wk; 1.5 g HMB-Ca	No	BIA	NS
Cabre et al. [95]	RCT; DB	Young to middle-aged untrained men and women; 32.2 ± 10.0 y; n = 15–25/group	Supervised high-intensity resistance training and high-intensity interval running 2 d/wk	Assessment	6 wk; 1.5 g HMB (type not specified) on training days only	50 mg caffeine; 550 mg choline; 25 g carbohydrate; 1500 IU vitamin D3; 15 g whey protein; 5 caseinate protein; 200 mg vitamin C, 45 IU D-alpha tocopherol; 1.5 g glucosamine	DXA	NS
Fairfield et al. [96]	RCT; DB	Middle-aged untrained women; 45–60 y; n = 9–10/group	Supervised progressive resistance training; 3 d/wk	Assessment	12 wk; 3 g/d HMB-Ca	2000 IU Vitamin D3	DXA (whole body) MRI (thigh)	DEXA: NS MRI: −28 cm/3 (*p* = 0.05)
Han et al. [97]	RCT; blinding not described	Older men and women with sarcopenia and hip fracture; ≥ 65 y; n = 43–45/group	Supervised progressive resistance training; every 2–3 days	No	3 months; 3 g/d HMB (type not specified)	No	DXA	NS

4C, 4-compartment; ATP, adenosine triphosphate; BIA, bioelectrical impedance analysis; BIS; bioimpedance spectroscopy; Ca, calcium; DB, double-blinded; DXA, dual-energy x-ray absorptiometry; FA, free acid; FFM, fat-free mass; FM, fat mass; HMB: β-hydroxy-β-methylbutyrate; IU, international units; LBM, lean body mass; MRI, magnetic resonance imaging; NS; no significant effect(s); RCT, randomized controlled trial; TOBEC, total body electrical conductivity.

Differences with p-values >0.05 are noted as findings only when reported as such by the researchers.

Where only graphical data were reported, significant changes were estimated from figures and indicated by “~” ahead of changes.

^a^Creatine co-supplementation in a separate group; results for combined supplementation not reported.

^b^Pre-post absolute values were not reported; unclear whether reported changes reflect change in percent body fat or percent change relative to baseline.

^c^A number of editorials have been submitted related to these studies [98, 99,100] and editorials have been responded to by the authors [101–103].

HMB supplementation has been shown to increase fat-free mass (FFM) in response to exercise training in previously untrained individuals in as little as three weeks [6]. Among previously trained individuals, however, benefits are more likely to be realized over longer duration training protocols (i.e. ≥6 weeks) as short-term studies in trained individuals often do not detect benefits of HMB over placebo, although there are some exceptions [58,79]. It appears that there is even a benefit of HMB to increase FFM in athletes [104].

Interpretations of body composition changes are somewhat limited in most studies due to the lack of dietary control, especially habitual protein intake. However, most studies instructed participants not to change their diets and included some form of dietary assessment, typically a diet recall. A few studies tightly controlled participants’ diets [81,85], and these studies were notably associated with some of the largest magnitude of changes observed to date in body composition with HMB supplementation during training. These studies were questioned for the large effects reported.

Due to dual mechanisms of enhancing protein synthesis and reducing protein breakdown, HMB may uniquely support optimal body composition during training and caloric restriction, which is often practiced by athletes participating in weight-classified sports. In a very small study (*n* = 4/group) of female judo athletes who were calorically restricted (mean intake of ~1160 kcal/d, 1.33 g/kg protein/d) for three days, the group taking HMB experienced significant reductions in body fat percentage and numerically smaller reductions in FFM compared to the control group [105]. In another study with energy restriction, researchers evaluated the effects of HMB supplementation on body composition during 8 weeks of resistance training and time-restricted feeding [91]. Though the intent-to-treat analysis did not show a definitive benefit of HMB on body composition, in the per-protocol analysis, which excluded individuals who dropped out of the study or had poor diet, supplement, or exercise compliance, only the HMB group had significantly reduced fat mass and body fat percentage relative to baseline values, and had a numerically greater change in FFM.

Though most studies evaluating the effects of HMB supplementation have been conducted with young (primarily male) adults, the effects of HMB supplementation during exercise training have also been evaluated in different age groups [21,77,84,106,107]. In adolescent (aged 13–18 y) elite volleyball players, HMB supplementation during 7 weeks of training significantly increased lean mass and decreased fat mass [83]. To our knowledge, Fairfield et al. [96] were the first to specifically evaluate the effects of exercise and HMB in middle-aged women. Though no significant effects of HMB + exercise were observed for overall body composition, HMB + vitamin D_3_ supplementation significantly decreased intramuscular adipose tissue, as measured by magnetic resonance imaging of the thigh. Notably, a similar effect was observed in both resistance exercise trained women and concurrent sedentary control groups. Additional studies are needed to evaluate the metabolic or other health implications of these findings.

In older adults (aged ≥60 years), the influence of HMB during exercise training on body composition has been evaluated in several independent studies with mixed results. Eight weeks of supplementation with HMB during exercise training resulted in a tendency for increased lean mass and significantly reduced body fat percentage compared with placebo when measured using skinfolds. However, these measures were not significantly different between groups based on DXA analysis [77]. Notably, a significantly greater reduction in thigh fat area was observed using a sensitive computed tomography analysis, which aligns with the reduction in intramuscular fat observed with HMB supplementation in middle-aged women [96]. Two longer term studies (24 or 52 weeks) did not show any additional benefit of HMB supplementation during exercise training on overall body composition [21,84]. However, *post hoc* analysis revealed a greater reduction in abdominal adiposity among older men after 12 weeks of exercise with HMB supplementation compared to placebo [86]. The exercise training protocols utilized in most studies with older adults to date provided a smaller stimulus in terms of both volume and intensity than most studies conducted in younger age groups. Although the data are currently limited, HMB supplementation in combination with exercise in older adults continues to be an active area of research [108,109].

Jakubowski et al. [110] carried out a systematic review and meta-analysis to determine the efficacy of HMB supplementation in augmenting FFM and strength gains during resistance exercised training (RET) in young adults. The authors concluded that HMB increased total body mass gain, but this effect did not translate into significantly greater increases in FFM or strength or decreases in fat mass during periods of RET. The authors did draw attention to the mean difference between the HMB-supplemented and placebo-supplemented groups of 0.29 kg (95% CI −0.01, 0.60, *p* = 0.06). This meta-analysis raised several statistical concerns, including the use of strong statements of conclusions that appear inconsistent with limitations of the analysis and a lack of adequate justification for decisions and descriptions of methods used in analysis (inadequate transparency and reproducibility). Jakubowski et al. [110] report the exclusion of two of the studies (Kraemer at al. [81] and Wilson et al. [85]) late in the analysis. The exclusions were not based on *a priori* inclusion/exclusion criteria and therefore should require extra justification and the conclusions be viewed with caution. Further, Jakubowski et al. included their own study [111] that was not placebo-controlled; a requirement for inclusion in the meta-analysis. Lastly, a number of studies or individual trials were missing from the meta-analysis [6,7,56] which may have changed the FFM conclusions of the report.

In summary, HMB supplementation, when combined with exercise training, likely improves measures of body composition. This improvement is achieved through an increase in FFM and/or a decrease in fat mass. The benefits of improved body composition have been observed in most, but not all, of the studies conducted across participants of different ages, sexes, and baseline training statuses. HMB supplementation may also protect FFM and augment fat loss in athletes during mild-to-moderate dietary restriction, though additional studies in this area are warranted. It is worth noting that the most significant improvements in body composition with HMB have been observed in studies that have incorporated robust resistance training programs and dietary control. However, it is important to highlight that these factors are not necessarily required to experience the purported benefits of HMB. Due to the diversity in study populations and designs, it is recommended to critically evaluate the results of individual studies based on their suitability for the target population and training stimulus.

## 9. The effect of HMB on strength and power when combined with training

Increased recovery with HMB supplementation may allow athletes to train harder and more frequently resulting in faster training adaptations and greater increases in muscle strength and power (Table 2). However, the effects on strength and power have varied based on training status.

**Table 2.** Studies combining HMB with resistance training on exercise performance in untrained, mixed train/untrained and trained subjects. ⇓ = no change compared to placebo, ⇔ = numerical but not statistically significant change compared to placebo, ⇑ = significant increase compared to placebo (* *p* < 0.05 vs placebo).

Author	Subjects	Exercise	Supplementation and Study Length	Results Strength + Power
***Untrained***				
Nissen et al. [6]	41 untrained males (19–29 yrs.)	3 weeks of resistance training, 1.5 h 3 days per week.	1.5 g or 3 g HMB-Ca or placebo for 3 weeks.	⇑ total weight lifted*
Gallagher et al. [7]	37 untrained men (21.7 ± 1.0 y)	10 different exercises performed 3× per week at 80% (1RM).	3 g or 6 g HMB-Ca or placebo for 8 weeks.	⇑ isometric and isokinetic strength* ⇔ 1RM whole body strength
Asadi et al. [112]	16 untrained males (21.4 ± 0.7 y)	6-week (12 sessions) with each lasted 70–80 min. (1RM) bench press and leg press and vertical jump prior to and after training intervention.	3 g HMB-FA or placebo for 12 weeks.	⇑ greater improvement in estimated peak power output, increases in 1RM leg press, and increases in body mass*
***Untrained and trained***				
Panton et al. [58]	43 male, 41 female trained + untrained weightlifters (20–40 y)	4 weeks of resistance training 3 times per week.	3 g HMB-Ca or placebo for 4 weeks.	⇑ upper body strength*
***Trained***				
Nissen et al. [6]	32 trained collegiate male football players (19–22 y)	7 weeks of resistance and aerobic training, 2–3 h/d, 6d/week.	3 g HMB-Ca or placebo for 7 weeks.	⇑ upper body strength*
Kreider et al. [19]	40 resistance-trained athletes (25.1 ± 1.1 y)	4 weeks of resistance training.	3 or 6 g HMB-Ca or placebo for 4 weeks.	⇔ dose-dependent non-statistically significant increase in strength
Slater et al. [76]	17 water polo and rowing athletes (24.7 ± 1.6 y)	2–3 resistance exercises per week measuring 3-repetition maximum isoinertial strength.	3 g HMB-Ca or placebo for 6 weeks.	⇓ no significant changes
Ransone et al. [78]	35 collegiate American football players (21.3 ± 1.2 y)	4 hours per day for 4 days each week muscular strength, including bench press, squats, and power cleans.	3 g HMB-Ca or placebo for 4 weeks with 1 week washout.	⇓ no significant changes.
Thomson et al. [113]	22 resistance trained men (24.6 ± 4.0 y)	(1RM) lower body (leg extension) and upper body (bench press, bicep preacher curl).	3 g HMB-Ca or placebo for 9 weeks.	⇑ lower body strength* ⇔ upper body strength
Hung et al. [105]	8 collegiate female judo athletes (21.5 ± 0.9 y)	V.O_2max_ on a treadmill.	3 g HMB-Ca or placebo for 3 days.	⇔ muscle mass and the anaerobic performance of the lower and upper body were unchanged.
Portal et al. [83]	15 male, 14 female elite, national team level Israeli junior volleyball players (13.5–18 y)	18–22 hours of volleyball per week during first 7 weeks of season.	3 g HMB-Ca or placebo for 7 weeks.	⇑ increase 6RM bench press and leg press*. ⇑ greater knee flexion strength*. ⇑ increase in peak and mean anaerobic power by the Wingate anaerobic test*.
^a^Wilson et al. [85]	20 resistance trained males (21.6 ± 0.5 y)	Phase 1: 8-week-periodized resistance-training program. Phase 2: 2-week overreaching cycle. Phase 3: 2-week taper.	3 g HMB free acid (HMB-FA) or placebo for 12 weeks.	⇑ increased total strength (bench press, squat, and deadlift combined)* ⇑ greater increase in vertical jump power*.
Teixeira et al 2018 [13,90]	40 resistance-trained males (18–46 y).	8-week resistance training program (3 days/wk.)	3 g/d of either HMB-Ca, HMB-FA, or placebo.	⇔ no changes in upper and lower body strength or power
Durkalec-Michalski et al. [88]	58 trained male athletes (wrestling *n* = 12, judo *n* = 10, Brazilian jiu-jitsu *n* = 14, karate *n* = 6, rowing *n* = 16 (22 ± 6 y).	Athletes maintained daily training and dietary regimens.	3 g HMB-Ca or placebo for 12 weeks with 1 week washout.	⇑ increased aerobic capacity and anaerobic peak power*.

**Note**: ^a^A number of editorials have been submitted related to these studies [98–100] and editorials have been responded to by the authors [101–103].

### 9.1. HMB plus exercise in untrained subjects

Supplementation of 1.5 and 3 g/d of HMB-Ca has been shown to significantly increase total strength in untrained individuals following three weeks of resistance training in a dose-dependent manner [6]. Additionally, HMB-Ca supplementation reduces markers of muscle damage and muscle protein breakdown, which corresponded to improved muscle function [6]. Moreover, the effects of HMB-Ca on recovery in untrained individuals were confirmed when supplementation was combined with an eight-week resistance training program [7]. In the study, untrained subjects who supplemented with HMB-Ca showed increased isometric and isokinetic strength compared to placebo, however, exercise-induced changes in whole body strength failed to increase statistically over training alone (placebo +32.5%, 3 g/d HMB + 43.5%, and 6 g/d HMB + 45.5%) [7]. In a 6-week resistance training program, supplementation with HMB-FA increased peak power output (9.5%) and 1RM leg press (41.3%), compared to placebo (0.4%, and 25.3% respectively) [112]. Furthermore, HMB-FA supplementation significantly improved adrenocorticotropic hormone (ACTH) and cortisol responses to training, along with associated improvements in GH and IGF-1 training effects [112].

### 9.2. HMB plus exercise in mixed untrained and trained subjects

The effects of HMB-Ca were examined in male and female cohorts with four weeks of resistance training, three days per week. The cohorts consisted of both untrained and trained participants. The results showed no significant differences in strength gains based on prior training status or gender when HMB-Ca supplementation was used. The combined data demonstrated that HMB-Ca, regardless of gender or training status, increased upper body strength and reduced muscle damage compared to the placebo [58].

### 9.3. HMB plus exercise in trained subjects

Nissen et al. [6] were the first to describe the effects of HMB-Ca in trained athletes. In a 7-week study, the HMB-Ca supplemented group increased bench press by 7.5% compared to a 1.7% increase in the placebo group. This was accompanied by a significant improvement in FFM. However, the addition of 0, 3, or 6 g/d HMB-Ca in a randomized double-blind manner to 40 experienced resistance trained athletes for 28 days who maintained their individualized training program failed to significantly increase 1RM strength vs. control treated-athletes [19]. In addition, HMB-Ca supplementation failed to increase strength beyond the strength increases observed when a placebo was taken in either elite male water polo players and rowers during 6-weeks of 2–3 full-body resistance training sessions weekly [76]. Similarly, strength and body composition changes in a crossover, placebo-controlled design in 35 Division 1 college football players were similar when a placebo or 3 g/d of HMB-Ca were provided over a 4-week off-season resistance training program [78].

Studies of longer duration (over six weeks) in trained participants have shown that HMB supplementation may provide performance benefits. For example, a study involving nine weeks of HMB-Ca supplementation found an increase in lower body strength, though there was no effect on upper body strength [113] compared to placebo supplementation. Another study administered HMB-Ca supplementation (3 g/day) to elite volleyball players for seven weeks and observed greater increases in muscle strength and anaerobic properties, with no impact on aerobic capacity [83]. In a 12-week three-phase double-blind placebo-controlled study, HMB-FA supplementation in resistance-trained men resulted in an 18% increase in total strength (bench press, squat, and deadlift combined) and a 19% increase in vertical jump power during 8 weeks of periodized resistance training, followed by two weeks of overreaching and two weeks of tapering [85]. These changes were significantly higher than the 6% and 12% increases observed in the placebo group. Additionally, during the overreaching phase, HMB-FA supplementation helped maintain total strength, whereas the placebo group experienced a 4.5% loss. HMB-FA also reduced markers of muscle damage compared to the placebo group [85]. Notably, this paper has been criticized in the scientific literature [98] for reporting training outcomes that, based upon other nutritional and pharmaceutical interventions, were challenging to practically accept and for its statistical reporting, although the authors did publish a response to these criticisms [101]. While these results have sparked much contention, this paper has been included to allow the reader to understand and draw their own conclusions about the entire body of scientific literature involving HMB supplementation. In contrast, an 8-week study found no effects of HMB-FA and HMB-Ca on body composition, muscle strength, and power in trained participants [13,90]. However, it is worth noting that these studies did not show any effects of the training program on whole body, arm, or leg lean body mass in any of the groups, suggesting that the training program may not have been sufficient to increase muscle mass. Furthermore, total training volume was not reported, which would have helped substantiate whether adequate training progression occurred, and the supplements were not administered in a double-blind manner.

In summary, HMB has been shown to enhance strength and power in untrained individuals. However, the performance benefits of HMB in trained athletes are varied and tend to become more noticeable with longer study durations (over six weeks). Additionally, HMB’s effects on performance may be attributed to improved recovery and a decrease in muscle protein breakdown.

## 10. The effects of HMB on aerobic performance

While typically perceived as a supplement that enhances anaerobic exercise, HMB has also gained interest for its potential to improve aerobic performance in athletes. Although the precise mechanism remains unclear, HMB may enhance aerobic metabolism by upregulating the expression of PGC-1α, the master regulator of mitochondrial biogenesis, and activating AMPK kinase and Sirt1 [114,115]. This process could potentially promote mitochondrial biogenesis in both muscle cells and adipocytes, leading to improvements in carbohydrate and fat metabolism efficiency, increased oxygen consumption, and reduced fat mass [36,116,117]. Additionally, HMB may help attenuate muscle damage, thereby accelerating recovery. Knitter et al. [57] demonstrated the beneficial effects of HMB-Ca supplementation combined with training on muscle damage in runners. The HMB group exhibited significantly lower levels of muscle damage markers (decreased creatine kinase and lactate dehydrogenase response, two markers of muscle damage) following a 20-km run than the placebo group.

Several studies have investigated the effects of HMB supplementation on aerobic performance. Vukovich and Dreifort [118] studied the administration of 3 g/day of HMB-Ca in endurance-trained cyclists for 14 days. They found that while it did not impact maximal oxygen consumption (VO_2_peak), it significantly increased the onset of blood lactate accumulation (OBLA), which was defined as the VO_2_ corresponding to a blood lactate at 2 mm [119], potentially benefiting endurance performance. Lamboley et al. [80] investigated HMB-Ca combined with five weeks of treadmill interval training. They reported a 15.5% increase in maximal oxygen consumption (VO_2_Max) and a 13.4% increase in respiratory compensatory point (RCP) for the HMB-Ca supplemented group and an 8.5 and 8.6 % increase in the placebo group, respectively. Robinson et al. [120] studied HMB-FA with high-intensity interval training (HIIT), and observed higher increases in VO_2_peak and VT but no improvement in RCP compared to the placebo group following four weeks of HIIT. Durkalec-Michalski and Jeszka [121] administered HMB-Ca to highly trained combat athletes, and found a significant increase in VO_2_max and VT compared to the placebo group. Ferreira et al. [122] examined elite kayakers taking a daily HMB-Ca or placebo supplement for 14 days subjected to a maximum test of 4 min in a kayak ergometer. They found improved mechanical efficiency and higher levels of pulse generated, average power, and stroke frequency with HMB-Ca supplementation compared to a placebo. The study suggested that HMB supplementation may help kayakers generate more power, improving their performance.

HMB-Ca has also been shown to increase markers of endurance performance in experienced, competitive combat athletes, including wrestlers, judokas, Brazilian jiu-jitsu practitioners, karate athletes, as well as rowers [88]. In a 12-week cross-over study, supplementation with 3.75 g/d of HMB-Ca increased maximal oxygen uptake, time to reach ventilatory threshold (VT), threshold load at VT, and the threshold heart rate at VT. This indicates an increase in aerobic capacity and also anaerobic peak power by approximately 11% compared to 1.7% in the placebo-supplemented group [88]. HMB showed no benefits on power performance in elite female judo athletes where HMB-Ca was provided (3 g/d) for three days during a period of caloric restriction, potentially due to the short duration of supplementation [105].

In summary, the aforementioned studies provide evidence to suggest that supplementation with HMB has the potential to enhance aerobic performance, particularly in trained athletes. The molecular mechanisms underlying the effects of HMB are unknown. Additional research is needed to determine the optimal dosage and duration of HMB supplementation required to achieve improvements in aerobic performance. Furthermore, future studies should explore the potential synergistic effects of combining HMB with other supplements, such as creatine monohydrate (CrM), as well as its impact on different athletic populations engaged in high-intensity and aerobic training modalities.

## 11. HMB supplementation in older adults

After the age of 40, LBM decreases at a rate of about 8% per decade [123–125] and accelerates to about 15% per decade after the age of 70 [124]. The underlying causes of this loss appear to be multifactorial [126,127] including maintaining a sedentary lifestyle, malnutrition, insulin resistance, oxidative stress, and alterations in skeletal muscle and protein metabolism. These factors contribute to the decrease in the muscle protein synthetic response and subsequent anabolic resistance often observed in the elderly [128–130]. It is suggested that anabolic resistance may be overcome by supplementation of leucine, and it has been hypothesized that this may be due to the conversion of leucine to HMB [131]. However, endogenous HMB is produced at low levels [132] in the body from leucine [133,134]. Previous work by Kuriyan et al. [135] demonstrated that plasma HMB concentrations vary with age and, in healthy adults, plasma HMB concentrations are positively associated with appendicular lean mass and hand grip strength. In a recent study that included 1290 older adults (74.6 ± 6.0 years), plasma HMB levels were inversely correlated with frailty and mainly with parameters related to body composition and strength [131]. Molina-Baena et al. [131] concluded that these findings suggest that HMB levels below a certain threshold are associated with frailty and weakness, while above a certain threshold, there is a protective effect. While reporting on outcomes from a large number of people, the correlative nature of these outcomes requires readers to avoid the temptation to draw cause-and-effect conclusions from this design and study results. Certainly, these associations are intriguing and warrant deeper investigation using well-controlled, more rigorous study designs.

The clinical benefits of HMB with and without exercise on skeletal muscle mass, strength, and functionality in adults ≥60 years have been extensively reviewed [136–144]. A recent umbrella review by Phillips et al. [143] indicates that HMB-Ca alone or part of a nutritional supplement are supportive of increases in lean soft-tissue mass (a proxy for muscle) retention and muscle strength in older adults. It was further concluded that additional studies may be needed to access the impact of HMB on muscle function [143]. These findings are confirmed by recent meta-analyses confirming the positive effects of HMB-Ca on muscle strength [142,145] and lean body mass [146,147] in older adults.

Stout et al. [84] showed an improvement in leg strength and muscle quality in adults aged ≥65 years with HMB-Ca supplementation compared to a placebo group, but these effects were not seen when combined with resistance exercise. In two randomized double-blind studies, Flakoll et al. [148] found that supplementation with HMB-Ca and two amino acids, arginine and lysine (HMB/Arg/Lys), for 12 weeks significantly increased muscle strength and improved functionality and tended to enhance the gain of muscle mass. These effects were attributed to increased whole-body protein synthesis. In a year-long study by Baier et al. [22], HMB/Arg/Lys significantly improved LBM in supplemented older adults but showed no improvements in muscle strength or function. A retrospective analysis of the same data [149] revealed that the benefit on muscle strength was highly dependent on circulating levels of Vitamin D (25-hydroxy-Vitamin D ≥30 ng/ml, 25OH-D). These findings suggest that adequate Vitamin D levels may be necessary to achieve optimal benefits with HMB. In a recent 12-month study, HMB-Ca plus Vitamin D_3_ resulted in increased serum 25-OHD levels and was associated with an improvement in LBM, strength, and muscle function in non-exercising older adults but no treatment effect differences were observed between the exercising groups [21]. However, Yang et al. [150] reported that HMB-Ca supplementation while undergoing resistance exercise training demonstrated an improvement in muscle strength, functionality, and muscle quality compared to placebo-supplemented older adults with sarcopenia.

In summary, HMB supplementation may be useful even in a non-exercise setting to improve muscle strength, functionality, and muscle quality, especially in older adults. The effects of HMB supplementation with exercise are varied, but the combination may have beneficial effects for the treatment of age-associated sarcopenia, frailty and cachexia, though this may be dependent on Vitamin D status [167].

## 12. HMB and muscle disuse atrophy

In addition to the deleterious effects of aging on muscle mass and strength, many older adults, even healthy individuals, experience periods of inactivity due to illness or injury. These periods of muscle disuse can result in rapid losses of muscle mass and strength [151]. Data from a well-controlled experimental bed rest study suggest that HMB supplementation can prevent the deleterious effects of bed rest in otherwise healthy older adults [152]. Over 10 days of experimental bed rest in 19 older adults, daily supplementation with 3 g of HMB, starting five days prior to bed rest, led to better maintenance of overall LBM compared to a placebo group (HMB: −0.17 ± 0.19 kg vs. PLA: −2.05 ± 0.66 kg, *p* = 0.02). Similar results were noted for leg lean mass (HMB: −0.08 ± 0.17 kg vs. PLA: −1.01 ± 0.35 kg, *p* = 0.02). Furthermore, after eight weeks of post-bed-rest rehabilitation and following a resistance training program for 3 days per week, the group supplemented with HMB tended to increase LBM relative to baseline values (0.71 ± 0.33 kg, *p* = 0.06), while the control group values remained below baseline (−0.06 ± 0.22 kg, *p* = 0.78). Similar patterns were observed for isokinetic muscle strength.

Analyses of muscle biopsy samples collected from these participants revealed higher levels of mitochondrial content and fusion/fission markers, as well as higher triglyceride levels in the muscle from individuals supplemented with HMB [153]. Meanwhile, muscle samples from the control group showed upregulated collagen synthesis and downregulation of genes associated with mitochondrial energetics. However, these effects were attenuated in the muscle from individuals supplemented with HMB [115]. These results support the modulation of mitochondrial dynamics and lipid metabolism by HMB as a potential mechanism for preventing disuse atrophy and aiding rehabilitation, going beyond HMB’s effects on rates of protein synthesis and degradation.

Other studies evaluating HMB supplementation during periods of relative disuse or tissue trauma (e.g. during recovery from surgery) have yielded similar results. Differences in maintenance of muscle cross-sectional area after total knee arthroplasty in older patients (aged 65–80 years) were not statistically significant different from each other (*p* > 0.05), but a group supplemented with 2.4 g HMB +14 g Glutamine +14 g Arginine had larger absolute changes (9.1 ± 16.6%) when compared to control (−3.6 ± 33.8%). In terms of muscle strength, the group supplemented with HMB did successfully attenuate the losses in muscle strength 14 days after surgery compared to the effects in the were observed in the control group [154]. Among older female patients with hip fracture, 81.3% of patients taking an HMB-containing supplement were ambulating by days 15 and 30 after surgery vs 26.7% of patients in the control group. Muscle strength was also significantly higher in the HMB vs the control group by day 30 after surgery [155]. HMB supplementation also showed better protection of muscle mass and strength compared to the control group at three months after hip replacement in a separate study of sarcopenic patients with hip fracture [97].

In summary, HMB supplementation may be useful for attenuation the loss of muscle mass and strength because of disuse atrophy in older adult populations or following surgical intervention and tissue trauma. However, the study data are limited, and additional research will be needed especially in younger populations.

## 13. Efficacy of HMB in combination with other nutrients

There are a number of studies demonstrating the additive or synergistic effect when HMB is combined with other nutrients. These effects may result from combining nutrients that act through different mechanisms, aid in absorption, or provide a limiting nutrient required to maximize exercise performance.

### 13.1. HMB plus probiotics

In athletes, supplementation of certain probiotic strains has been shown to increase nutrient absorption, exercise performance [156,157], and recovery from muscle-damaging exercise [158]. The probiotic *Weizmannia coagulans* GBI-30, 6086 (BC30) has been shown to increase amino acid absorption from animal and plant proteins [159,160]. In a separate study, when this probiotic strain was combined with HMB-Ca and HMB-FA, the combination improved peak HMB levels by 16% and total HMB exposure by 19% following supplementation [161]. Moreover, HMB-FA supplementation during intense military training significantly reduced inflammation, and co-administration with BC30 for four weeks synergistically improved the beneficial effects on muscle integrity [162] and the anti-inflammatory effects of IL-10. The beneficial effectiveness of co-administering BC30 with HMB may either be a result of the increased exposure of HMB to tissue and plasma, or due to the anti-inflammatory and anti-oxidant synergistic properties of the probiotic strain. More research is needed to fully explore these ideas.

### 13.2. HMB plus creatine

Creatine is involved in the recycling of adenosine triphosphate (ATP) and is primarily found in skeletal muscle and brain tissue. Creatine supplementation increases intramuscular creatine stores and acute exercise training capacity, resulting in greater gains in LBM, regional muscle size (thickness), strength, and athletic performance [163]. A total of nine studies have investigated the effects of co-administering HMB with creatine on athletic performance (Table 3) [56,79,92,164–170] and three of those studies employed a 2 × 2 factorial design, which allows for the examination of the additive and synergistic effects of HMB-Ca and creatine [56,79,92]. The first study examining potential added effects used a 3-week progressive resistance-exercise training program [56]. The additive effects of HMB-Ca and creatine supplementation were demonstrated by significant increases in LBM and accumulative muscle strength with no interaction [56]. Creatine plus HMB significantly increased aerobic power in elite male rowers compared to HMB-Ca or creatine alone [92]. Furthermore, HMB plus creatine supplementation, along with an intensive strength training program, increased anaerobic power, endurance, and improved body composition compared to HMB alone [79,170]. However, three studies showed no effect of combined HMB and creatine supplementation on exercise performance [164,165,168], while the individual ingredients (HMB [164,165], creatine [168]) showed no effect either.

**Table 3.** Summary of studies that investigated the effects of HMB plus creatine (CrM) on athletic performance in young adults.

Author	Subjects	Supplementation and Study Length	Results		
			HMB	CrM	HMB+CrM
Kreider et al. [170]	41 NCAA Division I football Players	HMB-Ca, or HMB-Ca + CrM or placebo for 4 weeks	⇔ Fat-free Mass		⇑ Fat-free mass
Almada et al. [169]	41 NCAA Division I football Players	HMB-Ca, or HMB-Ca + CrM or placebo for 4 weeks	⇑ Work Output ⇑Strength		⇑ Work Output ⇑Strength
Jówko et al. [56]	40 healthy males (21.0 ± 2.1 years)	HMB-Ca or CrM or HMB-Ca + CrM or placebo for 3 weeks	⇑ strength (1-RM)**	⇑ strength (1-RM)**	⇑ strength (1-RM)**
Zajac et al. [79]	52 well trained basketball players (25.6 ± 5.6 y)	CrM or HMB-Ca or CrM + hMB-Ca or placebo for 30 days	⇑ Fat-free mass* ⇓decrease body fat*	⇑ fat free mass*, maximal, total work*	⇑ fat free mass*, maximal, total work*^,^**** ⇓decrease body fat*
O’Connor et al. [165]	27 male elite rugby players (18–32 y)	HMB-Ca or HMB-Ca + CrM or placebo for 6 weeks	⇔ anaerobic or aerobic capacity		⇔anaerobic or aerobic capacity
O’Connor et al. [164]	30 male elite rugby players (24.9 ± 1.5 y)	HMB-Ca or HMB-Ca +3 g CrM or placebo for 6 weeks	⇔ strength, endurance, leg power.		⇔ strength, endurance, leg power.
Faramarzi et al. [166]	24 soccer players (21.6 ± 0.1 y)	HMB-Ca or HMB-Ca + CrM or placebo for 6 days	⇔mean and peak power		⇑mean power**, peak power**^,^****
Fernández-Landa et al. [92]	28 elite male rowers (30.43 ± 4.65 y)	CrM or HMB-Ca or CrM+ HMB-Ca or placebo for 10 weeks	⇑power output**	⇑power output**	⇑power output**^,^ ***^,^****
Mangine et al. [168]	16 collegiate (Division 1-AA) American rugby players (21.1 ± 1.6 y)	HMB-Ca or HMB-Ca + CrM for 6 weeks		⇔body composition, strength and sprinting kinetics	⇔body composition, strength and sprinting kinetics

**Note**: (*p < 0.05 vs baseline, **p < 0.05 vs. placebo, ***p < 0.05 vs. CrM. ****p < 0.05 vs. HMB).

In a recent study, Fernández-Landa et al. [92] examined the effect of CrM alone or in combination with HMB in 28 elite male rowers. Before and after the 10-week intervention, the researchers measured the power in wattage (W) of each rower at the anaerobic threshold (WAT), 4 mmol (W4), and 8 mmol (W8) of blood lactate using an incremental rowing ergometer test. At W8, CrM-HMB demonstrated a greater capacity than the placebo group, CrM, and HMB. The aerobic power at WAT, W4, and W8 showed a synergistic improvement when combining CrM and HMB.

### 13.3. HMB plus amino acids

L-arginine is a non-essential amino acid and has multiple functions, some related to protein synthesis and breakdown [171]. It is converted to nitric oxide, which causes blood vessels to dilate and enhances blood flow to the exercising muscle [172]. L-lysine is an essential amino acid that plays an important role in normal growth and muscle turnover [173]. The amino acid taurine has anti-inflammatory and antioxidant properties and plays a role in numerous physiological processes relevant to athletic performance, such as lipid and glucose regulation and energy metabolism [174]. L-glutamine may have beneficial effects on the immune system and glucose regulation during periods of intense training [175].

Daily supplementation with HMB-Ca, L-arginine, and L-lysine for 12 weeks increased strength, functionality, and muscle mass in elderly women without exercise [148], and supplementation for 12 months confirmed the benefits on muscle mass in elderly men and women [22]. However, the increase in muscle mass did not translate to increases in strength in people who were deficient in Vitamin D [149]. Co-administration of HMB-Ca with L-arginine, L-glutamine, and taurine significantly increased training-induced changes in LBM, muscle strength, and power in response to a 12-week resistance exercise training program compared to exercise alone [81]. The multi-ingredient HMB-Ca formula has also been shown to benefit immunomodulation in response to acute and chronic resistance exercise [176]. While the combined ingestion of HMB-Ca with L-arginine, L-glutamine, and taurine or the co-administration of HMB and L-lysine are effective compared to placebo, it is currently unknown if the multi-ingredient combinations are superior to HMB alone due to a lack of comparative studies.

### 13.4. HMB plus vitamin D_3_

Vitamin D is important for normal muscle development and optimizing strength and athletic performance, particularly in people with low vitamin D levels [177]. However, data on athletes is limited, with mixed results. Co-administration of HMB-Ca with vitamin D_3_ in healthy older adults with proven vitamin D insufficiency enhances muscle strength and physical function even without an exercise program [21]. The beneficial effects on muscle health were confirmed in a study using a fortified yogurt containing HMB-Ca, vitamin D_3_, and vitamin C for 12 weeks in elderly subjects, showing significant increases in strength [178]. Supplementation with HMB and vitamin D_3_ for 12-weeks increased muscle mass in sedentary middle-aged women. However, when supplementation was combined with progressive resistance exercise training, no additional benefits on muscle mass and functionality were observed compared to the exercise alone [96]. Ingesting HMB and vitamin D_3_ as part of a multi-ingredient blend further containing caffeine and choline in conjunction with a training program resulted in increased upper and lower body strength compared to a control group in an untrained adult population [95]. The added or even synergistic benefits of co-administering HMB or vitamin D_3_ cannot currently be established on any outcomes as comparative studies with HMB and vitamin D_3_ alone are currently lacking. Additional studies in younger athletic populations are needed to confirm the benefits seen in an older population.

### 13.5. HMB plus protein

Dietary protein is being proposed to optimize exercise training and recovery, and amino acid composition, especially leucine content, and the rate of digestion are considered markers of protein quality [179]. HMB has been shown to increase the anabolic effects of plant (soy) protein ingestion in a fasting catabolic state [180]. Combining HMB with isomaltulose and whey protein improved power and recovery from intense resistance exercise compared to whey protein alone [181]. Kreider et al. [19] administered a fortified protein/carbohydrate supplement with additional 0, 3, or 6 g/d HMB-Ca to resistance trained athletes for 28 days but showed no effect on body composition or strength. No differences between HMB-Ca, whey protein, or the combination of both were observed when examining exercise-induced declines in strength and markers of muscle damage [182]. Furthermore, Jakubowski et al. [111] compared the effects of HMB-Ca compared to leucine when added to whey protein during a 12-week phased resistance program and concluded that HMB-Ca added to whey protein did not show greater improvement in muscle mass or strength compared to leucine added to whey protein. However, the study was not placebo controlled.

### 13.6. HMB plus KIC or ATP

Co-administration of 3 g HMB-Ca with 0.3 g α-ketoisocaproic acid (KIC) for 14 days has been shown to reduce exercise-induced declines in performance and muscle damage [59]. In a study involving adolescent medium distance runners, supplementation of 3.75 g HMB-Ca and 10 g L-Arginine-ketoglutarate for 12 days prevented declines in jump performance during training without influencing markers of muscle damage [183]. However, HMB has also been shown to reduce muscle damage and enhance recovery in other studies. Another important factor in muscle health is adenosine-5′-triphosphate (ATP). Supplementation with ATP improves athletic performance by increasing blood flow to the exercising muscle, providing needed nutrients, and reducing fatigue [184,185]. When combined with resistance exercise training, ATP and HMB have been shown to increase lean body mass, power, and strength in comparison to control [186], as well as ATP [186] and HMB [85] alone. These publications [85,186] have been criticized by others [98–100], however, the authors addressed the methodological queries, current and past results, and provided additional data and information as requested [101–103]. Nonetheless, due to the criticism surrounding them, they were not used to inform the overall interpretation of the literature and the ISSN’s position statements.

In summary, the efficacy of HMB may be enhanced when combined with certain nutrients under select conditions. For instance, when HMB is co-administered with glutamine, arginine, and lysine, its effect on LBM may be increased. Similarly, when HMB is combined with creatine or ATP, it may also have a positive impact on LBM. Additionally, strength and function may be improved when HMB is used in combination with vitamin D (when vitamin D levels are insufficient), creatine, and ATP. Furthermore, the effectiveness of HMB in muscle recovery appears to be enhanced when co-administered with the probiotic BC30. However, HMB was not shown to always have an enhanced effect when combined with high levels of whey protein.

## 14. Final summary and conclusions

The following 12 points constitute the Position of the International Society of Sports Nutrition (ISSN). They have been approved by the Research Committee of the Society:

1. HMB is a metabolite of the amino acid leucine that is naturally produced in both humans and other animals. Two forms of HMB have been studied: Calcium HMB (HMB-Ca) and a free acid form of HMB (HMB-FA). HMB-FA appears to lead to increased appearance of HMB in the bloodstream when compared to HMB-Ca, though recent results are mixed.
2. The available safety and toxicity data suggest that chronic HMB-Ca and HMB-FA consumption are safe for oral HMB supplementation in humans up to at least one year.
3. There are no negative effects of HMB-Ca and HMB-FA on glucose tolerance and insulin sensitivity in humans. There may be improvements in glucose metabolism in younger adults.
4. The primary mode of action of HMB appears to be through its dual mechanism to enhance muscle protein synthesis and suppress muscle protein breakdown. HMB’s activation of mTORC1 is independent of the leucine-sensing pathway (Sestrin2-GATOR2 complex).
5. HMB may help reduce muscle damage and promote muscle recovery, which can promote muscle growth and repair. HMB may also have anti-inflammatory effects, which could contribute to reducing muscle damage and soreness.
6. HMB consumption in close proximity to an exercise bout may be beneficial to increase muscle protein synthesis and attenuate the inflammatory response. HMB can provide a beneficial physiological effect when consumed both acutely and chronically in humans.
7. Daily HMB supplementation at 38 mg/kg body weight in combination with exercise training may potentially improve body composition through increasing lean mass and/or decreasing fat mass with benefits in participants across age, biological sex, and baseline training status. The most pronounced of these improvements in body composition with HMB have been observed in studies with robust resistance training programs and dietary control.
8. HMB may improve strength and power in untrained individuals, but its performance benefits in trained athletes are mixed and increase with an increase in study duration (>6 weeks). Furthermore, HMB’s beneficial effects on athletic performance are thought to be driven by improved recovery.
9. HMB supplementation appears to potentially have a positive impact on aerobic performance, especially in trained athletes. The proposed mechanisms of the effects are unknown.
10. HMB supplementation may be important in a non-exercising sedentary and aging population to improve muscle strength, functionality, and muscle quality. The effects of HMB supplementation with exercise are varied, but the combination may have a beneficial effect on the treatment of age-associated sarcopenia under select conditions.
11. HMB may be effective in countering muscle disuse atrophy during periods of inactivity due to illness or injury. The modulation of mitochondrial dynamics and lipid metabolism by HMB may be a potential mechanism for preventing disuse atrophy and aiding rehabilitation beyond HMB’s effects on rates of muscle protein synthesis and degradation.
12. The efficacy of HMB in combination with certain nutrients may be enhanced under select conditions.
